# Species Substitution and Changes in the Structure, Volume, and Biomass of Forest in a Savanna

**DOI:** 10.3390/plants13192826

**Published:** 2024-10-09

**Authors:** Kennedy Nunes Oliveira, Eder Pereira Miguel, Matheus Santos Martins, Alba Valéria Rezende, Juscelina Arcanjo dos Santos, Mauro Eloi Nappo, Eraldo Aparecido Trondoli Matricardi

**Affiliations:** Department of Forest Engineering, Federal University of Brasília (UnB), Brasilia 70297-400, DF, Brazil; edermiguel@unb.br (E.P.M.); matheus.martins.28@gmail.com (M.S.M.); alba.rezende@gmail.com (A.V.R.); juscelina.santos@unb.br (J.A.d.S.); mauronappo@yahoo.com.br (M.E.N.); ematricardi@unb.br (E.A.T.M.)

**Keywords:** Cerrado, Cerradão, forest inventory, diversity, increment, dynamics

## Abstract

Research related to Cerradão vegetation focuses more on the floristic-structural aspect, with rare studies on the quantification of volume and biomass stocks, and even fewer investigating the increments of these attributes. Using a systematic sampling method with subdivided strips and 400 m^2^ plots, the density found was 1135, 1165, and 1229 trees/ha in 2012, 2020, and 2023, respectively, in Lajeado State Park, Tocantins State, Brazil. Volume was estimated using the equation $v=0.000085D^{2.122270}H^{0.666217},$ and biomass was estimated using the equation $AGB=0.0673{\left(\rho D^{2}H\right)}^{0.976}$. Vegetation dynamics were assessed using growth increment, recruitment, mortality, turnover rate, and time. The results indicated that dynamics have increased since the start of monitoring. Typical Cerrado species, in the strict sense, were replaced by those from forest environments. The total production in volume and biomass was 160.91 m^3^/ha and 118.10 Mg/ha, respectively, in 2023. The species of *Emmotum nitens*, *Mezilaurus itauba*, *Ocotea canaliculata*, and *Sacoglottis guianensis* showed the highest increment values in volume and biomass. For the community, the average values were 4.04 m^3^/ha/year and 3.54 Mg/ha/year. The community has not yet reached its carrying capacity and stores a significant amount of biomass. This is influenced by the transition of the study area from an exploited environment to a conservation unit (park) and by its location in a transitional area with the Amazon biome.

## 1. Introduction

The world’s forests cover approximately 4.06 billion hectares, accounting for about one-third of the Earth’s surface. These forests provide habitat for 80% of amphibian species, 75% of bird species, and 68% of mammal species. Tropical forests are particularly notable, as they are home to around 60% of all vascular plant species worldwide [1].

In terms of extent, the Cerrado (savanna) is the second-largest biome in Brazil. Despite being recognized as a global biodiversity hotspot with a high degree of endemism and risk of degradation [2,3,4], it is estimated that between 40 and 50% of its original cover has been lost, about 19.8% of the original cover remains untouched [5,6,7], and only 3% is protected within strict protection units [8,9]. Among the world’s tropical ecosystems, the Cerrado is seen as one of the last great terrestrial frontiers, with intense land use aimed at expanding intensive agriculture to meet the demand for agricultural products in Brazil and globally. In contrast, it features dynamic landscapes, high amounts of biodiversity, and the headwaters of important Brazilian river basins [10]. This biome harbors a vast diversity of vascular plants, totaling more than 12,000 species [11], and is considered the most biodiverse savanna, with about 35% of its flora composed of endemic species, representing about 1.5% of the world’s endemic plant species [12,13]. It consists of 11 physiognomic types, including forest, savanna, and grassland formations. The forest formations of the Cerrado encompass riparian forests, gallery forests, dry forests, and Cerradão [14].

Among these formations, Cerradão stands out negatively as it occurs in only 1% of the biome [15], and is associated with interfluve areas, well-drained terrains, and deep soils of the Latosol (predominantly) and dystrophic Cambisol classes [16]. The trees have an average height ranging from 8 to 15 m, with continuous canopies that cover 50 to 90% of the ground. From a physiognomic perspective, Cerradão is a forest; however, from a floristic standpoint, it resembles Cerrado sensu stricto (c.s.s.) [14,17]. The coexistence of trees and shrubs over a matrix of herbs and grasses is the main characteristic of the grassland and savanna formations of the biome [10], while forest formations have few or no grasses.

The analysis of biological variables in frequency categories provides insights into their behavior [18]. In nature, different distribution patterns can be identified, including unimodal, multimodal, and negative exponential or decreasing patterns. The negative exponential pattern is observed in natural or native forests, characterized by continuous regeneration [19]. Describing this aspect helps determine if populations can be considered sustainable [20], meaning they include individuals with the potential to migrate from smaller to larger diameter classes.

The growing interest in structure and dynamics, and especially in quantifying forest volume and biomass in tropical climates, reflects the importance of these variables’ stocks in these ecosystems [21,22,23,24,25,26]. Forest dynamics encompass a variety of processes occurring at distinct spatial and temporal scales, from stomatal opening and closure (minutes to days, at the leaf level) to biome changes (decades to centuries, spanning entire continents) [27]. Dynamics include processes such as the recruitment, growth, mortality, and turnover of individuals within the forest community [28].

For Cerrado vegetation, precise estimates of production and productivity for volume and biomass stocks are still very rare and limited. This is primarily due to the large diversity of species, the variability among individuals of the same species, and the wide range of trunk and canopy forms among individuals. Generally, most research focusing on volumetric production estimates has been concentrated on forest formations [29].

In the specific case of Cerradão, research related to vegetation focuses more on floristic aspects (including composition, richness, and species diversity) and descriptions of vegetation structure [30], with extremely rare studies on quantifying volume and biomass stocks, and even fewer investigating the increment of these attributes, either by species or for the community.

Therefore, we assess changes in the structure, volume, and biomass of tree vegetation in a Cerradão area located in Lajeado State Park, Tocantins state, Brazil.

## 2. Results

### 2.1. Phytosociology, Floristics, and Diversity

In 2012, we sampled 1135 trees/ha, distributed across 34 botanical families and 69 species. Among the identified families, the richest in number of species were Fabaceae, with 13 species, and Chrysobalanaceae, Malpighiaceae, and Melastomataceae, each with four species. The most numerous families, with more than 100 trees/ha, were Myrtaceae (189 trees), Melastomataceae (129 trees), Annonaceae (116 trees), and Fabaceae (101 trees). On the other hand, the families of Araliaceae, Connaraceae, Opiliaceae, Bignoniaceae, Combretaceae, and Proteaceae had only one tree per hectare, thus they were considered rare in the community. The species with the highest number of trees per hectare were *Myrcia fenzliana* (189 trees), *Miconia albicans* (106 trees), and *Xylopia aromatica* (100 trees), followed by *Emmotum nitens* (84 trees).

In 2020, the floristics were quite similar to those recorded in 2012. We sampled 1165 trees/ha, distributed across 34 botanical families and 69 species. The richest families in terms of species were Fabaceae, with 14 species, and Chrysobalanaceae, Malpighiaceae, and Melastomataceae, each with 4 species. Regarding tree density, the most numerous families, with more than 100 trees/ha, were Myrtaceae (158 trees), Melastomataceae (130 trees), Annonaceae (124 trees), and Lauraceae (116 trees). A total of seven families had only one tree per hectare (Araliaceae, Dilleniaceae, Proteaceae, Salicaceae, Bignoniaceae, Combretaceae, and Connaraceae). The species with the highest number of trees per hectare were *Myrcia fenzliana* (154 trees), *Emmotum nitens* (98 trees), and *Miconia albicans* (94 trees).

In 2023, we recorded 1229 trees/ha, distributed across 35 botanical families and 70 species. The richest families in terms of species were Fabaceae, with 14 species, and Chrysobalanaceae and Malpighiaceae, each with 4 species. Regarding tree density, the most numerous families, with more than 100 trees/ha, were Myrtaceae (144 trees), Lauraceae (134 trees), Melastomataceae (130 trees), Annonaceae (114 trees), and Metteniusaceae (112 trees). Eight families had only one tree per hectare, the same as in the previous monitoring, with the addition of Elaeocarpaceae. The species with the highest density were *Myrcia fenzliana* (135 trees), *Emmotum nitens* (112 trees), and *Tapirira guianensis* (104 trees).

**Figure 1:**
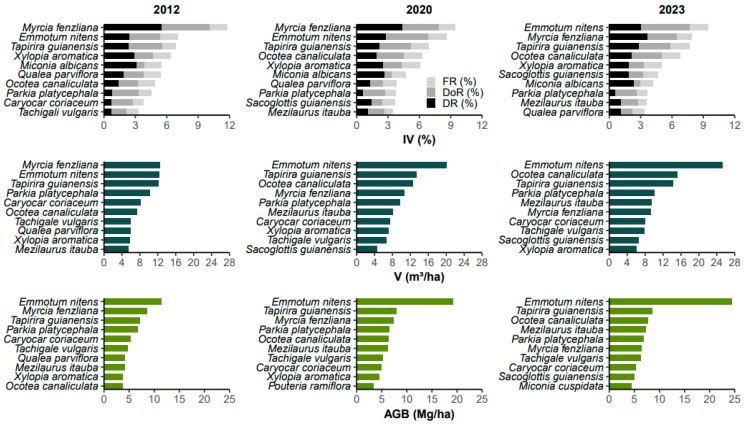
Importance Value (IV%), volume (V), and aboveground biomass (AGB) of the top 10 species according to IV% for the years of 2012, 2020, and 2023 in the Cerradão of Lajeado State Park, Tocantins, Brazil. FR is relative frequency; DoR is relative dominance; and DR is relative density.

When ordering the species in descending order according to the Importance Value (IV %), in 2012, the top ten species represented about 59%, a percentage that dropped to around 56% in 2020 and 2023. *Emmotum nitens*, *Myrcia fenzliana*, and *Tapirira guianensis* together account for an average of 25% of the IV (%) (Figure 1).

Five populations showed a decrease in their importance in the community. In the first period (2012–2020), *Caryocar coriaceum* and *Tachigali vulgaris* dropped out of the group of the ten (10) most important species based on IV%. *Myrcia fenzliana* represented just over 12% of the IV% in 2012, a value that fell to around 9% and less than 8% in 2020 and 2023, respectively. *Miconia albicans* and *Qualea parviflora* remained in the group of the most important species during the monitoring periods, but with a reduction in their values. These species are typical of Cerrado sensu stricto (c.s.s.), except for *M. fenzliana*, which is typical of gallery forests [14]. Conversely, *Emmotum nitens*, *Mezilaurus itauba*, *Ocotea canaliculata*, and *Sacoglottis guianensis* increased their importance consecutively in the three surveys studied.

Regarding diversity, on average, throughout the study period (2012–2023), the area had a Shannon–Weiner index (H′) of 3.28 and a Pielou’s evenness index (J′) of 0.77. Pielou’s evenness changed little between inventories (0.76–0.78), and the H′ values in 2012 (3.22) and 2020 (3.31) differed according to Hutcheson’s *t*-test (t = −2.76; *p* < 0.05), possibly due to the long eight-year interval. Additionally, the establishment of the park in the study area and the initiation of conservation activities may have influenced the results. The H′ values in 2020 (3.31) and 2023 (3.33) did not differ according to the same test (t = 0.59; *p* > 0.05), and Pielou’s evenness remained unchanged between inventories (0.78) (Table 1).

**Figure 2:**
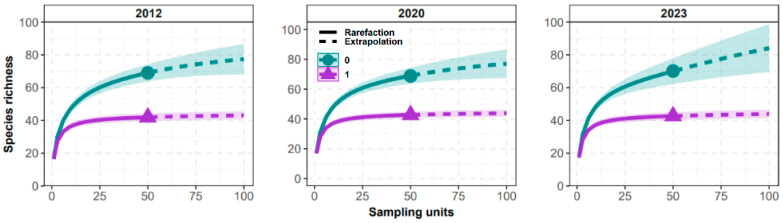
Accumulation curve of richness and diversity as a function of the number of sampling units in the Cerradão of Lajeado State Park, Tocantins, Brazil. Guides = 0 and 1, where guides refer to the Hill number “q” (0 = species richness and 1 = Shannon–Wiener diversity) [32]. Rarefaction (solid line) and extrapolation (dashed lines) correspond to mean values and standard deviation ranges with confidence intervals (α = 0.05).

The study of a plant community in space and time can be represented by the relationship between species richness and sampling units through accumulation curves [31] and interpolation using permutation algorithms (e.g., bootstrap). In the last year surveyed (2023), the community showed a species richness (S) of 70 species, with the potential to reach just over 80 species on average. However, achieving this would require doubling the current sampling effort (Figure 2).

### 2.2. Horizontal and Vertical Structure

**Figure 3:**
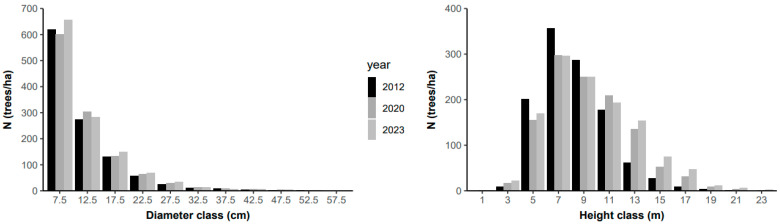
Distribution of the frequency of individuals (N) in the arboreal community of the Cerradão located in Lajeado State Park, Tocantins, Brazil.

The distribution of individuals across diameter classes followed a J-reverse or negative exponential distribution model, with most individuals in the smaller classes. According to the Kolmogorov–Smirnov test (KS), the distribution of individuals across diameter classes did not differ between the 2012–2020 inventories (KS, D = 0.18; *p* > 0.05) and the 2020–2023 inventories (KS, D = 0.09; *p* > 0.05). The first diameter class (7.5 cm) on average accounts for ~53% of all sampled trees. Approximately 25% of the trees are in the second diameter class (12.5 cm), and only 2.8%, on average, have a diameter greater than 30 cm (Figure 3). If the forest does not exhibit this common structure, it may indicate that some disturbance has affected the vegetation.

Regarding height classes, the distribution of individuals also did not differ between the 2012–2020 inventories (KS, D = 0.17; *p* > 0.05) and the 2020–2023 inventories (KS, D = 0.08; *p* > 0.05). Most individuals are found in the intermediate classes (7 and 9 m) (Figure 3). On average, over 80% of the trees have heights ranging from 5 to 11 m, and only 1.1% of the trees reach heights greater than 19 m. The classes of 13–17 m have seen an increase in the number of individuals since the beginning of the monitoring period.

### 2.3. Aboveground Volume and Biomass

The results of the ANOVA indicated significant differences between the years (volume: F_(2,147)_ = 5.67, *p* < 0.01; aboveground biomass: F_(2,147)_ = 7.38, *p* < 0.01). Tukey’s test revealed that the volume in 2012 differed significantly from the volume in 2023 (*p* < 0.05), but there were no significant differences between the volumes in 2012 and 2020 (*p* = 0.09) and between the volumes in 2020 and 2023 (*p* = 0.45). Regarding biomass, that of 2012 differed significantly from that of 2020 (*p* < 0.05) and from that of 2023 (*p* < 0.05), while there was no significant difference between the biomass of 2020 and 2023 (*p* = 0.35).

**Figure 4:**
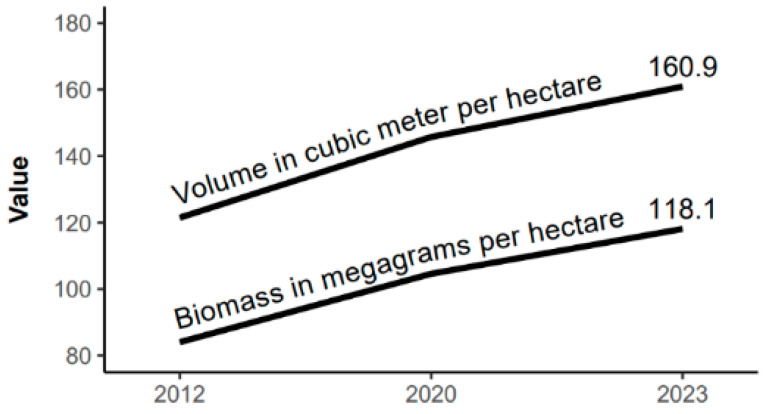
Volume and aboveground biomass production of the arboreal vegetation in Cerradão over time in Lajeado State Park, Tocantins, Brazil.

The volumetric production of the area was 121.47, 145.75, and 160.91 m^3^/ha for the years of 2012, 2020, and 2023, respectively. For biomass, the values were 84.40, 104.60, and 118.10 Mg/ha, respectively, for the mentioned years (Figure 4). In the latest monitoring, the volume, aboveground biomass production, and the Importance Value (IV %) highlighted *Emmotum nitens* as the most important species, with IV = 9.43%, V = 25.27 m^3^/ha (~15% of the total community volume) and AGB = 24.47 Mg/ha (~20% of the total community biomass). On average, for the community during the monitored period, there was an increase of 2–3% per year in volumetric production and 3–4% per year in aboveground biomass (Figure 4).

**Figure 5:**
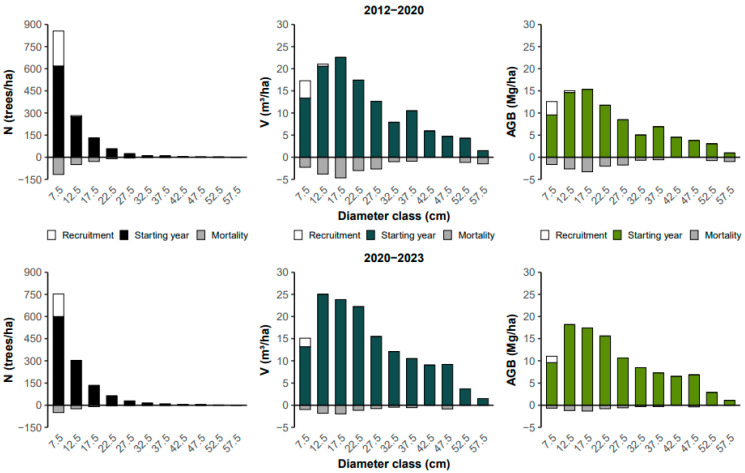
Changes in the woody community by diameter class of the Cerradão in Lajeado State Park, Tocantins, Brazil. N is the number of trees; V is volume; and AGB is the aboveground biomass.

The diameter class range of 12.5–27.5 cm produced, on average, nearly 60% of the volume per hectare over the three monitored years (Figure 5). In terms of biomass, this same diameter range corresponded to 35.7% and 34.0%, respectively, during the periods of 2012–2020 and 2020–2023. The reduction in the contribution of these classes was mainly due to the increase in production from the next class (22.5 cm), which rose from 11.8 Mg/ha to 15.6 Mg/ha in the respective periods.

**Figure 6:**
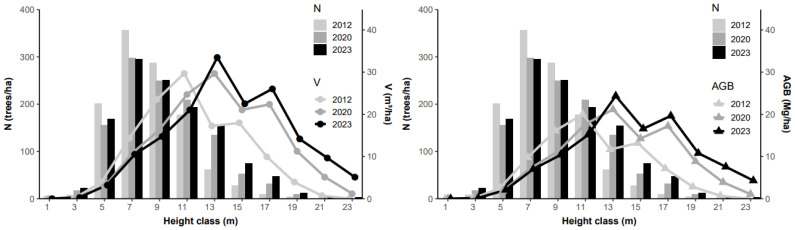
Volume (V) and aboveground biomass (AGB) of the woody vegetation in the Cerradão of Lajeado State Park, as a function of height class.

Regarding biomass, approximately 50% (53.5% in 2012, 51.8% in 2020, and 47.9% in 2023) of the per hectare production falls within the height class range of 11–15 m. The 13 m class exhibited the highest aboveground biomass production in 2020 (21.15 Mg/ha) and 2023 (24.36 Mg/ha), a position held in 2012 by the 11 m class at 20.02 Mg/ha (Figure 6).

### 2.4. Dynamics

For the studied area (2.0 ha), we sampled 2269, 2330, and 2457 trees in the years of 2012, 2020, and 2023, respectively. Among the inventories from 2012 to 2020, only one species was absent (*Ferdinandusa elliptica*), and one was included (*Inga cylindrica*). In the period from 2020 to 2023, one species was absent (*Mouriri glazioviana*), but two were included (*Connarus perrottetii* and *Sloanea guianensis*). Out of the total sampled trees in the second monitoring period, 489 were recruited and 428 died (approximately 61 and 54 trees/ha/year, respectively), resulting in an annual recruitment rate (R) of 2.90% and an annual mortality rate (M) of 2.58%. In the last monitoring period, these values were 307 recruited trees and 180 deaths, approximately 102 and 60 trees/ha/year recruited and dead, respectively, resulting in a recruitment rate (R) of 4.30% per year and a mortality rate (M) of 2.64% per year. On average, the mortality rate (M) for the studied Cerradão area was 2.60% per year, and the recruitment rate (R) was 3.63% per year.

Keeping the current recruitment rate, the time required for the community to double its size was 24.23 years from 2012 to 2020 and 16.30 years from 2020 to 2023. A shorter doubling time indicates a more dynamic community. Following the same reasoning, it will take about 26.2 years on average (26.53 years from 2012 to 2020 and 25.86 years from 2020 to 2023) for the community to halve its size, maintaining the current mortality rate (M).

For turnover time, 23 years was the average value found for the Cerradão community studied in Lajeado State Park. The average annual turnover rate in number of trees (Trot), which expresses the overall dynamics of populations [33], was 3.12% per year (2.74% per year from 2012 to 2020 and 3.49% per year from 2020 to 2023) (Table 2).

The 10 species that led in volumetric production and biomass in the latest monitoring account for approximately 70% of the total volume and biomass over the entire period. Similar to what was observed for the Importance Value (IV%), excluding the species *Emmotum nitens*, due to its clear prominence in the community, the species *Mezilaurus itauba* (0.38 m^3^/ha/year and 0.31 Mg/ha/year), *Ocotea canaliculata* (0.75 m^3^/ha/year and 0.39 Mg/ha/year), and *Sacoglottis guianensis* (0.52 m^3^/ha/year and 0.40 Mg/ha/year) stood out by presenting the highest values of Annual Periodic Increment (IPA) for both volume and aboveground biomass (Table 3 and Table 4). For the community, the values of Annual Periodic Increment in volume (IPA_V_) and aboveground biomass (IPA_AGB_) were 4.04 m^3^/ha and 3.54 Mg/ha, respectively, of which 3.09 m^3^/ha and 3.00 Mg/ha are solely from the top ten (10) species in these attributes, based on the latest monitoring (2023).

## 3. Discussion

### 3.1. Phytosociology, Floristics, and Diversity

During the monitored period, there was a floristic replacement of species typical of the Cerrado sensu stricto (c.s.s.) by species from forest formations, possibly indicating a successional advance. Importance Value (IV) is an essential tool in the structural analysis of the community; however, according to this index, the most important species may or may not be those with the highest volume and/or aboveground biomass production.

Despite the changes in species richness and floristic composition, the values of Shannon’s Index (H′) and Pielou’s evenness (J′) changed little between the monitored periods, indicating a certain stability of the Cerradão community in terms of diversity and species distribution. Similar values have been found when studying Cerradão areas in the Central-West [34,35] and Southeast regions of Brazil [36]. In Cerradãos across six Brazilian states, Shannon’s index ranged from 3.1 to 4.0 and evenness ranged from 0.79 to 0.83 [16]. A higher J value indicates a more uniform distribution of species within the sample or community [37]. Therefore, the studied arboreal Cerradão community exhibits a uniform dispersion and high species diversity, with an H′ value (3.28) close to the upper limit of commonly found values.

Fabaceae is a prominent family in species richness in the Cerradão of Lajeado State Park. Moreover, it is worth noting that the central region of Brazil is recognized as the main center of diversity for this family [38], which is the third largest botanical family in the world in terms of species number [39]. The clear difference in floristic richness between Fabaceae and other families, observed in this study and in other works in the Cerrado [40] is possibly related to the family’s ability to fix nitrogen, which is a highly advantageous characteristic in environments with dystrophic soils [41], such as those found in the studied Cerradão.

The curve that expresses richness as a function of the sampled area shows rapid initial growth as new species are added, but as an increase takes place in the number of sample units, it tends to stabilize, indicating that most species in the community have been identified, and thus, the sampling was sufficient.

### 3.2. Horizontal Structure and Vertical Structure

From the diameter structure, it is possible to observe that the arboreal community of Cerradão in Lajeado State Park can be considered sustainable, meaning it includes individuals with the potential to migrate from smaller to larger diameter classes, showing a J-reverse or negative exponential distribution, common in primary forests.

It is noteworthy that, on average, more than one third of the individuals had intermediate height, and there was a significant increase in the number of individuals in the 13–17 m height classes. These changes in structure contributed to the high volume and aboveground biomass production.

### 3.3. Volume and Aboveground Biomass

ANOVA and the Tukey test indicated significant differences in the production of volume and aboveground biomass between the monitored years. For the observed total means, eight years were sufficient to detect differences in aboveground biomass production (2012–2020), while eleven years were sufficient to detect differences in volumetric production (2012–2023).

Lajeado State Park features some species similar in size to those found in the Amazon rainforest, which elevates volumetric and biomass values. It holds biomass stocks higher than those found in other Cerradão areas (30–62 Mg/ha) [21,30,42,43,44]. A study conducted in Cerradão areas in the Nhecolândia Pantanal, Brazil, using the formula proposed by Brown et al. [45] for tropical forests, found around 97.88 Mg/ha for tree biomass [46]. These results highlight the study area’s capacity to accumulate biomass.

The state of Tocantins is located in the geographical transition zone between the Cerrado and the Amazon rainforest [47]. Clements [48] first applied the term “ecotone” to describe the transition area where two ecosystems meet and interact. The Cerrado–Amazon Transition (CAT) is a vast ecotonal region spanning over 6000 km, situated between the two largest biomes in South America [47]. It represents the largest frontier zone between savanna areas, represented by the Cerrado (the most extensive and diverse savanna), and forest, represented by the Amazon (the largest tropical forest in the world) [49]. Therefore, the geographical location factor is crucial for the study and understanding of volume and aboveground biomass production in the Cerradão of Lajeado State Park.

Intermediate diameter classes increased their share in the total volume and biomass (Figure 5). Due to the increase in the number of individuals in the mentioned height class (item 3.2), there was biomass accumulation at higher heights, accompanied by an increase in total biomass between measurements, indicating that the arboreal vegetation of the Cerradão in Lajeado State Park continues to grow and accumulate biomass.

### 3.4. Dynamics

The intensity of community dynamics has increased since the beginning of monitoring. Turnover time is the arithmetic average between half-life and doubling time, with more intense community dynamics indicated by lower turnover values [19]. A higher turnover rate and lower turnover time reflect a more dynamic period. Therefore, among the monitored time intervals, the second period (2020–2023) was the most dynamic.

There was mortality and recruitment mainly in the first diameter classes (Figure 5). On average, typical values for annual mortality rates in primary tropical forests range from 1 to 2% per year [50]. More abundant species are subject to higher mortality and recruitment rates due to their high population density and, especially, their sensitivity to dynamics [51]. As diameters increase, it is expected that mortality and recruitment rates will decrease [40]. Thus, despite the park being established only in 2001 [52], this area has a mortality rate similar to that of primary forests.

In response to the positive disparity between average annual mortality and recruitment rates, it was observed that the half-life time exceeded the doubling time for the community as a whole. This phenomenon represented a pattern of positive transformations over the studied period.

Regarding the annual increment in volume (IPA_V_) and aboveground biomass (IPA_AGB_) of the top 10 species in the last year (2023), only two are typical of Cerrado sensu stricto (c.s.s.), such as *Caryocar coriaceum* (marginal areas of Cerrado to the north-northeast) and *Tachigali vulgaris* [14,53]. Species of c.s.s. are giving way to species more typical of forest environments, which showed higher growth rates compared to other species in the community, indicating a successional advance.

## 4. Materials and Methods

### 4.1. Study Area

**Figure 7:**
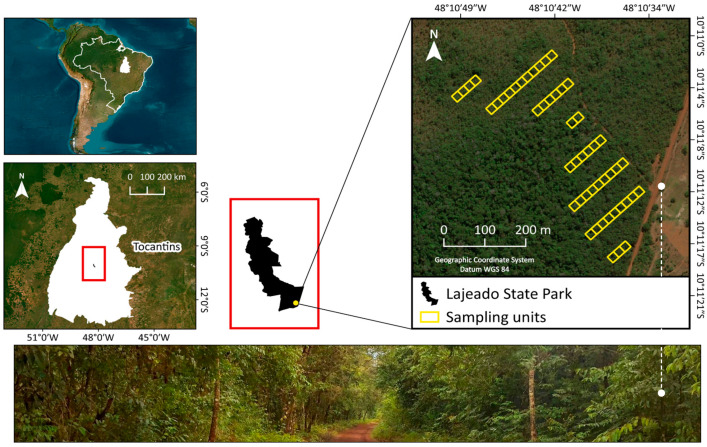
The distribution of sampling units (50) and the location of the study area in the Cerradão of Lajeado State Park, Tocantins State, Brazil.

This study was conducted in Lajeado State Park (PEL), in the state of Tocantins, Brazil, in an area of Cerradão spanning approximately 10 hectares, located between parallels 10°10′55″ and 10°11′20″ south latitude, and between meridians 48°10′50″ and 48°10′30″ west longitude (Figure 7). The soil of the studied Cerradão is classified as dystrophic (pH between 4.0 and 4.8; Al^3+^ > 1.3 cmol_c_/kg; Ca^2+^ < 0.4 cmol_c_/kg) [54].

Including water balance and climatic indices, the predominant climate in the region is classified as C2wA’a’, characterized by humid and subhumid conditions with moderate water scarcity during winter [55,56]. This climate is marked by two distinct seasons, with a dry period from May to September and a rainy season from October to April. The state of Tocantins exhibits notable longitudinal variation (from east to west) in annual precipitation, ranging from 1300 to 1900 mm, along with an opposite change in rainfall seasonality from west to east [57]. The average monthly precipitation for the study area is around 100 mm [58].

The lowest average temperatures occur in June and July (~24 °C), coinciding with the driest months of the year. The highest average temperatures occur in September (28.4 °C) (28.4 °C) [58], a period when rainfall is also low (~50 mm) [59].

### 4.2. Forest Inventory

**Figure 8:**
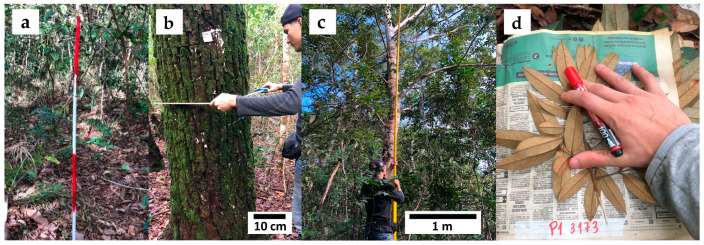
(**a**) Stakes for demarcat ion of sampling units; (**b**) Use of caliper for diameter measurements; (**c**) Graduated pole for height measurement and estimation; and (**d**) Collection of botanical material for subsequent identification and herbarium preparation.

In 2012, a forest inventory was implemented in the Cerradão area using a systematic sampling process [60] in strips 20 m wide and variable in length, subdivided into plots of 20 m × 20 m (400 m^2^). In total, eight strips were established, spaced 60 m apart, with 50 plots, covering a total area of two hectares (Figure 7). The corners of each plot were marked (Figure 8a), aiming for the continuous monitoring of the vegetation.

The diameter at 1.3 m above the ground (D) and the total height (H) of all living and standing dead trees with D ≥ 5 cm were recorded. Diameter was measured using a caliper (Figure 8b), and height was measured using a graduated 15 m pole (Figure 8c). Heights greater than 15 m were estimated visually, using the pole as a reference. Trees with two or more stems had each stem with a D ≥ 5 cm measured, recording the diameter and height of each stem. All recorded trees were botanically identified at a species, genus, and family level using the APG IV classification [61]. Botanical identification of each tree, when possible, was carried out in the field, and botanical material from the trees was collected and herbarium specimens were prepared [62], aiming for later identification by area specialists (Figure 8d). The monitoring was conducted in the years of 2012, 2020, and 2023, covering a period of over a decade (11 years).

### 4.3. Data Analysis

#### 4.3.1. Floristics and Diversity

The floristics of the Cerradão were evaluated both by richness, represented by the number of species, genera, and families recorded in each monitoring period, and by species diversity using the Shannon–Wiener Index [63]. Generally, the Shannon–Wiener Index (H′) ranges between 1.5 and 3.5, occasionally exceeding 4.5. Opting for natural logarithms as the base, the mathematical properties of H′ demonstrate greater consistency and coherence. Therefore, the use of natural logs per individual [64] is not only recommended but also reflects a global trend towards using the natural base [63,65].

The relative proportion of species was also evaluated using Pielou’s Evenness Index (J′), which is a derivation of the Shannon–Wiener Diversity Index [66,67] (Table 5). This index reflects the degree of uniformity in the distribution of individuals among the different species present.

To assess significant differences in H′ occurrence among the three monitored periods (2012, 2020, and 2023), Hutcheson’s t-test was used, considering a significance level of 5% [68].

The sample sufficiency for species richness and diversity was further assessed using rarefaction and extrapolation curves, for q = 0 (species richness—S) and 1 (Shannon–Wiener Index—H′), where q represents a numeric value specifying the diversity order of Hill numbers [32]. Extrapolations were conducted from abundance data with 100 bootstrap replications and 95% confidence intervals [69], using Rstudio software version 4.3.0 [70].

#### 4.3.2. Structure of the Vegetation

##### Phytosociological Structure

The vegetation structure was evaluated based on phytosociological analysis, which considers the variable density, dominance, and frequency of species within the community, and consequently, the corresponding Importance Value Index (IV%). The IV% represents the ecological relevance level of each species in the local guild or assemblage [71] and is obtained by summing the relative values of density, dominance, and frequency for each species (Table 6).

Density indicates the proportion between the number of individuals of each species and the total number of individuals in the sampled community, per unit area [72]. Dominance is related to the degree of occupation of individuals of a species within the community and is represented by the variable basal area, obtained by the ratio of the basal area of a species to the total basal area of the sampled community per unit area. Finally, frequency is obtained from the ratio of the number of plots where a particular species was recorded to the total number of sampled plots. This variable reflects the average dispersion of a species in a community and the likelihood of encountering that species in a sample unit.

##### Diametric and Vertical Structure

The vegetation structure was also assessed based on the diameter distribution of sampled trees in each monitoring period, namely in 2012, 2020, and 2023. The diameter and height class intervals were maintained at 5 cm and 2 m, respectively, across all three monitoring periods. To assess significant differences between the distributions of trees by diameter class and height for the monitoring periods of 2012–2020 and 2020–2023, the Kolmogorov–Smirnov goodness-of-fit test [73] was used.

#### 4.3.3. Quantification of Aboveground Volume and Biomass Stocks

The volume for each tree was estimated using a specific volumetric equation for the Cerradão of the study area [74]:

(1) $$v=0.000085D^{2.122270}H^{0.666217}({R^{2}}_{ajus}=0.99;S_{yx}=0.023m^{3};S_{yx}\%=15.01)$$

where v is the total volume with bark per tree (m^3^), D is the diameter at 1.3 m above ground (cm), H is total height (m); R^2^_ajus_ is the adjusted coefficient of determination, S_yx_ is the standard error of the estimate, and S_yx_% is the standard error of the estimate expressed as a percentage.

The aboveground biomass for each tree was estimated using the pantropical model proposed by Chave and colleagues [75]. They conducted a regression analysis using tree biomass related to the product *ρ*D^2^H and identified, with 4004 trees sampled by rigorous volume measurement for adjustment, the following allometric equation:

(2) $$AGB=0.0673{\left(\rho D^{2}H\right)}^{0.976}$$

where AGB is aboveground biomass (kg), D is diameter (cm), H is height (m), and *ρ* is basic wood density (g/cm^3^).

This equation generally achieves an accuracy of 90% on a scale of 2500 m^2^ in humid tropical forest, with a mean bias of around 10%. As for the basic density of individuals, it was obtained from secondary databases [76,77,78,79]. The accuracy for most individuals was at the species level, though for some, it was only at the genus level (Table 7).

To investigate significant differences in total aboveground volume and biomass between the years of 2012, 2020, and 2023, a one-way Analysis of Variance (ANOVA) was performed. The data were transformed using the natural logarithm (ln), and the assumptions of normality and homoscedasticity were met. To identify which years differed from each other, Tukey’s multiple comparisons test was conducted.

#### 4.3.4. Vegetation Dynamics

The dynamics of the Cerradão tree vegetation were assessed over the monitored periods of 2012–2020 and 2020–2023. The evaluation considered the following components of dynamics: growth (increment) [80], rates of mortality and recruitment [81], turnover, half-life time, doubling time, and turnover time [82,83] (Table 8). Data from individual-level monitoring were used to calculate the annual periodic increment (IPA), both for the sampled community and for the top ten species by volume (IPA_V_) and aboveground biomass (IPA_AGB_), based on the latest inventory (2023).

## 5. Conclusions

The analysis of the arboreal community in the Cerradão at Lajeado State Park revealed an increase in dynamics since the beginning of monitoring. The park is undergoing a continuous process of ecological succession, characterized by the replacement of species typical of Cerrado sensu stricto with species from forest formations. Despite this floristic change, the community has maintained stability in terms of structure, diversity, and species distribution.

Regarding the volume and aboveground biomass, the park presented stocks superior to those found in other Cerradão areas, highlighting its high accumulation capacity. This fact is influenced both by the creation of the conservation unit, which transformed the study area into a non-exploited environment, and by its location in a transition zone bordering the Amazon biome. The Cerradão at Lajeado State Park has not yet reached its maximum carrying capacity and continues to store a significant amount of volume and aboveground biomass.

## Figures and Tables

**Table 1:** Species richness, abundance, diversity, and evenness parameters during the monitoring periods in the Cerradão of Lajeado State Park, Tocantins, Brazil.

Parameter	Monitoring Year		
	2012	2020	2023
Number of Species (S)	69	69	70
Number of Trees (trees/ha)	1135	1165	1229
Hmax * or ln(S)	4.23	4.23	4.25
Shannon–Wiener Index (H′)	3.22	3.31	3.33
Pielou’s Evenness Index (J′)	0.76	0.78	0.78

* Maximum diversity.

**Table 2:** Growth in volume and aboveground biomass and dynamic parameters related to the number of individuals in the woody community of the Cerradão in Lajeado State Park, Tocantins, Brazil.

Parameter	Period		Unit
	2012–2020	2020–2023	
Time Interval	8	3	years
Mortality	214	90	trees/ha
Recruitment	245	154	trees/ha
Mortality Rate (M)	2.57	2.64	%/year
Recruitment Rate (R)	2.90	4.34	%/year
Turnover Rate (T_rot_)	2.74	3.49	%/year
Half-life Time (t_½_)	26.53	25.86	years
Doubling Time (t_2_)	24.23	16.30	years
Turnover Time (t_rot_)	25.38	21.08	years
Absolute Growth in volume	24.28 ^ns^	15.16 ^ns^	m^3^/ha
Absolute Growth in aboveground biomass	20.56 *	13.50 ^ns^	Mg/ha
Volume growth rate	2.50	3.47	%/year
Aboveground biomass growth rate	3.06	4.30	%/year

Tukey test: ns indicates not significant; * indicates significant difference.

**Table 3:** Annual Periodic Increment in volume (IPA_V_) for the top 10 species in volumetric production based on the latest monitoring of the Cerradão area in Lajeado State Park, Tocantins, Brazil.

Species	Family	V (m^3^/ha)			IPA_V_ (m^3^/ha/Year)		Average
		2012	2020	2023	2012–2020	2020–2023	
*Emmotum nitens*	Metteniusaceae	12.33	20.07	25.27	0.97	1.73	1.35
*Ocotea canaliculata*	Lauraceae	7.40	12.58	15.15	0.65	0.86	0.75
*Tapirira guianensis*	Anacardiaceae	12.12	13.32	14.27	0.15	0.32	0.23
*Parkia platycephala*	Fabaceae	10.24	9.62	10.10	−0.08	0.16	0.04
*Mezilaurus itauba*	Lauraceae	5.54	8.06	9.36	0.32	0.44	0.38
*Myrcia fenzliana*	Myrtaceae	12.45	10.65	9.21	−0.23	−0.48	−0.35
*Caryocar coriaceum*	Caryocaraceae	8.23	7.51	7.99	−0.09	0.16	0.04
*Tachigali vulgaris*	Fabaceae	6.00	6.56	7.76	0.07	0.40	0.24
*Sacoglottis guianensis*	Humiriaceae	1.64	4.52	6.58	0.36	0.69	0.52
*Xylopia aromatica*	Annonaceae	5.83	7.10	6.02	0.16	−0.36	−0.10
Total (10 species)		81.78	99.97	111.72	2.27	3.92	3.09
Total (sampled)		121.47 ^A^	145.75 ^AB^	160.91 ^B^	3.03	5.05	4.04

Identical letters indicate that groups do not differ significantly according to Tukey’s test.

**Table 4:** Annual Periodic Increment in aboveground biomass (IPA_AGB_) for the top 10 species with the highest biomass production, based on the latest monitoring of the Cerradão area in Lajeado State Park, Tocantins, Brazil.

Species	Family	AGB (Mg/ha)			IPA_AGB_ (Mg/ha/Year)		Average
		2012	2020	2023	2012–2020	2020–2023	
*Emmotum nitens*	Metteniusaceae	11.46	19.28	24.47	0.98	1.73	1.35
*Tapirira guianensis*	Anacardiaceae	7.17	7.95	8.62	0.10	0.22	0.16
*Ocotea canaliculata*	Lauraceae	3.67	6.32	7.69	0.33	0.46	0.39
*Mezilaurus itauba*	Lauraceae	4.13	6.25	7.34	0.27	0.36	0.31
*Parkia platycephala*	Fabaceae	6.69	6.50	6.92	−0.02	0.14	0.06
*Myrcia fenzliana*	Myrtaceae	8.58	7.40	6.38	−0.15	−0.34	−0.24
*Tachigali vulgaris*	Fabaceae	4.66	5.26	6.36	0.07	0.37	0.22
*Caryocar coriaceum*	Caryocaraceae	5.31	4.97	5.29	−0.04	0.11	0.03
*Sacoglottis guianensis*	Humiriaceae	1.21	3.37	4.97	0.27	0.53	0.40
*Miconia cuspidata*	Melastomataceae	1.67	3.20	4.48	0.19	0.43	0.31
Total (10 species)		54.55	70.50	82.53	1.99	4.01	3.00
Total (sampled)		84.0 ^A^	104.6 ^B^	118.1 ^B^	2.57	4.50	3.54

Identical letters indicate that groups do not differ significantly according to Tukey’s test.

**Table 5:** Formulas used in the floristic analysis and diversity of tree vegetation of the Cerradão in Lajeado State Park, Tocantins, Brazil.

Variable	Formula
Shannon–Wiener Index (H′)	$H^{\prime }=-\sum_{i=1}^{N}\frac{n_{i}}{N}\cdot ln\left(\frac{n_{i}}{N}\right)$
Pielou’s Evenness (J′)	$J\prime =\frac{H\prime }{\ln(S)}$

N = total number of sampled trees; n_i_ = number of sampled trees of species i; S = total number of species; ln = natural logarithm.

**Table 6:** Formulas used in the phytosociological analysis of tree vegetation in the Cerradão of Lajeado State Park, Tocantins, Brazil.

Variable	Unit	Formula
Basal Area of species i (G_i_)	m^2^	$G_{i}=\sum_{i=1}^{n}\frac{\pi {D_{i}}^{2}}{40.000}$
Absolute Density of species i (DA_i_)	trees/ha	${DA}_{i}=\frac{n_{i}}{A}$
Relative Density of species i (DR_i_)	%	${DR}_{i}=\frac{{DA}_{i}}{\sum_{i=1}^{n}{DA}_{i}}\cdot 100$
Absolute Dominance of species i (DoA_i_)	m^2^/ha	${DoA}_{i}=\frac{G_{i}}{A}$
Relative Dominance of species i (DoR_i_)	%	${DoR}_{i}=\frac{{DoA}_{i}}{\sum_{i=1}^{n}{DoA}_{i}}\cdot 100$
Absolute Frequency of species i (FA_i_)	%	${FA}_{i}=\frac{P_{i}}{\sum_{i=1}^{n}P_{i}}\cdot 100$
Relative Frequency of species i (FR_i_)	%	${FR}_{i}=\frac{{FA}_{i}}{\sum_{i=1}^{n}{FA}_{i}}\cdot 100$
Importance Value of species i (IV_i_)	%	${VI}_{i}={DR}_{i}+{DoR}_{i}+{FR}_{i}$

D_i_ = Diameter at 1.30 m above ground level of tree i (cm); P_i_ = number of plots where species i occurs; A = Total sampled area; n_i_ = number of sampled trees of species i.

**Table 7:** Basic wood density (*ρ*) of tree species sampled in the Cerradão of Lajeado State Park, Tocantins, Brazil.

Species	*ρ*	Precision	Species	*ρ*	Precision
*Agonandra brasiliensis* Miers ex Benth. & Hook.f.	0.82	Species	*Moquilea egleri* (Prance) Sothers & Prance	0.64	Species
*Andira cordata* Arroyo ex R.T.Penn. & H.C.Lima	0.77	Genus	*Licania kunthiana* Hook.f.	0.88	Species
*Aspidosperma macrocarpon* Mart. & Zucc.	0.71	Species	*Leptobalanus octandrus* Sothers & Prance	0.76	Species
*Bocageopsis multiflora* (Mart.) R.E.Fr.	0.61	Species	*Mabea fistulifera* Mart.	0.64	Species
*Bowdichia* virgilioides Kunth	0.86	Species	*Machaerium acutifolium* Vogel	0.68	Species
*Terminalia tetraphylla* (Aubl.) Gere & Boatwr.	0.62	Species	*Maprounea guianensis* Aubl.	0.70	Species
*Byrsonima crassifolia* (L.) Kunth	0.58	Species	*Matayba guianensis* Aubl.	0.81	Species
*Byrsonima pachyphylla* A.Juss.	0.68	Species	*Mezilaurus itauba* (Meisn.) Taub. ex Mez	0.73	Species
*Byrsonima sericea* DC.	0.72	Species	*Miconia albicans* (Sw.) Steud.	0.69	Species
*Caryocar coriaceum* Wittm.	0.69	Genus	*Miconia cuspidata* Naudin	0.88	Species
*Casearia arborea* (Rich.) Urb.	0.57	Species	*Mouriri glazioviana* Cogn.	0.84	Genus
*Connarus perrottetii* (DC.) Planch.	0.55	Species	*Mouriri pusa* Gardner	0.84	Genus
*Copaifera langsdorffii* Desf.	0.65	Species	*Myrcia fenzliana* O.Berg	0.73	Species
*Cordiera sessilis* (Vell.) Kuntze	0.68	Species	*Myrcia splendens* (Sw.) DC.	0.80	Species
*Dalbergia miscolobium* Benth.	0.62	Species	*Ocotea canaliculata* (Rich.) Mez	0.48	Species
*Davilla elliptica* A.St.-Hil.	0.49	Species	*Ocotea nitida* (Meisn.) Rohwer	0.54	Species
*Didymopanax morototoni* (Aubl.) Decne. & Planch.	0.46	Species	*Ouratea hexasperma* (A.St.-Hil.) Baill.	0.63	Species
*Dimorphandra gardneriana* Tul.	0.79	Genus	*Parkia platycephala* Benth.	0.69	Species
*Diospyros sericea* A.DC.	0.60	Species	*Physocalymma scaberrimum* Pohl	0.85	Species
*Emmotum nitens* (Benth.) Miers	0.93	Species	*Plathymenia reticulata* Benth.	0.50	Species
*Eriotheca gracilipes* (K.Schum.) A.Robyns	0.47	Species	*Pouteria ramiflora* (Mart.) Radlk.	0.77	Species
*Eriotheca pubescens* (Mart.) Schott & Endl.	0.50	Species	*Protium heptaphyllum* (Aubl.) Marchand	0.63	Species
*Erythroxylum squamatum* Sw.	0.71	Species	*Qualea grandiflora* Mart.	0.61	Species
*Ferdinandusa elliptica* (Pohl) Pohl	0.65	Species	*Qualea parviflora* Mart.	0.73	Species
*Hancornia speciosa* Gomes	0.68	Species	*Roupala montana* Aubl.	0.78	Species
*Handroanthus serratifolius* (Vahl) S.Grose	0.92	Species	*Rourea induta* Planch.	0.47	Species
*Heteropterys byrsonimifolia* A.Juss.	0.61	Species	*Sacoglottis guianensis* Benth.	0.67	Species
*Himatanthus articulatus* (Vahl) Woodson	0.49	Species	*Simarouba versicolor* A.St.-Hil.	0.44	Species
*Hirtella glandulosa* Spreng.	0.93	Species	*Sloanea guianensis* (Aubl.) Benth.	0.82	Species
*Hymenaea stigonocarpa* Mart. ex Hayne	0.90	Species	*Tachigali vulgaris* L.G.Silva & H.C.Lima	0.74	Species
*Hymenolobium petraeum* Ducke	0.71	Species	*Tapirira guianensis* Aubl.	0.57	Species
*Inga alba* (Sw.) Willd.	0.61	Species	*Thyrsodium spruceanum* Benth.	0.64	Species
*Inga cylindrica* (Vell.) Mart.	0.48	Species	*Vatairea macrocarpa* (Benth.) Ducke	0.79	Species
*Kielmeyera coriacea* Mart. & Zucc.	0.56	Species	*Virola sebifera* Aubl.	0.65	Species
*Kielmeyera lathrophyton* Saddi	0.67	Species	*Vochysia gardneri* Warm.	0.38	Species
*Lafoensia pacari* A.St.-Hil.	0.80	Species	*Xylopia aromatica* (Lam.) Mart.	0.59	Species

**Table 8:** Formulas used in the analysis of tree vegetation dynamics in the Cerradão of Lajeado State Park, Tocantins, Brazil.

Variable	Unit	Formula
Mortality Rate (M)	%/year	M=1−N0−NmN01t·100
Recruitment Rate (R)	%/year	R=1−1−NrNt1t·100
Turnover Rate (Trot)	%/year	$T_{rot}=\frac{M+R}{2}$
Doubling Time (t2)	years	$t_{2}=\frac{ln(2)}{ln\left(1+\left(\frac{R}{100}\right)\right)}$
Half-life Time (t½)	years	$t_{½}=\frac{ln(0.5)}{ln\left(1-\left(\frac{M}{100}\right)\right)}$
Turnover Time (trot)	years	$t_{rot}=\frac{t_{2}+t_{½}}{2}$
Annual Periodic Increment (IPAY)	Y/year	${IPA}_{Y}=\frac{Y_{t}-Y_{0}}{t}$

N_0_ = initial number of live trees; N_m_ = number of trees that died during the period; N_t_ = number of live trees at the end; N_r_ = number of recruited trees; t = time interval (years); Y = volume or biomass; Y_0_ = Y value at the beginning of the period; Y_t_ = Y value at the end of the period.

## Data Availability

The data presented in this study are available upon request from the corresponding author.
